# Environmental temperature and growth faltering in African children: a cross-sectional study

**DOI:** 10.1016/S2542-5196(20)30037-1

**Published:** 2020-03-25

**Authors:** Lucy S Tusting, John Bradley, Samir Bhatt, Harry S Gibson, Daniel J Weiss, Fiona C Shenton, Steve W Lindsay

**Affiliations:** a Department of Disease Control, London School of Hygiene & Tropical Medicine, London, UK; b MRC Tropical Epidemiology Group, London School of Hygiene & Tropical Medicine, London, UK; c Big Data Institute, Nuffield Department of Medicine, University of Oxford, Oxford, UK; d Department of Infectious Disease Epidemiology, Imperial College London, London, UK; e Department of Biosciences, Durham University, Durham, UK

## Abstract

**Background:**

Child growth faltering persists in sub-Saharan Africa despite the scale-up of nutrition, water, and sanitation interventions over the past 2 decades. High temperatures have been hypothesised to contribute to child growth faltering via an adaptive response to heat, reduced appetite, and the energetic cost of thermoregulation. We did a cross-sectional study to assess whether child growth faltering is related to environmental temperature in sub-Saharan Africa.

**Methods:**

Data were extracted from 52 Demographic and Heath Surveys, dating from 2003 to 2016, that recorded anthropometric data in children aged 0–5 years, and were linked with remotely sensed monthly mean daytime land surface temperature for 2000–16. The odds of stunting (low height-for-age), wasting (low weight-for-height), and underweight (low weight-for-age) relative to monthly mean daytime land surface temperature were determined using multivariable logistic regression.

**Findings:**

The study population comprised 656 107 children resident in 373 012 households. Monthly mean daytime land surface temperature above 35°C was associated with increases in the odds of wasting (odds ratio 1·27, 95% CI 1·16–1·38; p<0·0001), underweight (1·09, 1·02–1·16; p=0·0073), and concurrent stunting with wasting (1·23, 1·07–1·41; p=0·0037), but a reduction in stunting (0·90, 0·85–0·96; p=0·00047) compared with a monthly mean daytime land surface temperature of less than 30°C.

**Interpretation:**

Children living in hotter parts of sub-Saharan Africa are more likely to be wasted, underweight, and concurrently stunted and wasted, but less likely to be stunted, than in cooler areas. Studies are needed to further investigate the relationship between temperature and child growth, including whether there is a direct effect not mediated by food security, regional wealth, and other environmental variables. Rising temperature, linked to anthropogenic climate change, might increase child growth faltering in sub-Saharan Africa.

**Funding:**

UK Medical Research Council and UK Global Challenges Research Fund.

## Introduction

Childhood undernutrition, including growth faltering and micronutrient deficiencies, is associated with increased mortality and is estimated to contribute to approximately 45% of deaths of children younger than 5 years globally.^1^ Despite reductions in stunting, wasting, and underweight in nearly all African countries between 2000 and 2015, the prevalence of stunting remains as high as 30% in some parts of sub-Saharan Africa.^2^ The WHO Global Nutrition Targets for 2012 to 2025 aim to reduce the prevalence of stunting by 40% and the prevalence of wasting to less than 5%. Many countries in sub-Saharan Africa are on track to reach these targets by 2025, but few are expected to achieve Sustainable Development Goal 2.2—to end malnutrition by 2030.^3^

Growth faltering is commonly attributed to prenatal, genetic, and epigenetic factors, as well as environmental exposures after birth, including inadequate diet, unclean water and poor sanitation and hygiene, poor parental education, repeat acute episodes of disease, and chronic subclinical inflammation of the small intestine.^4^ Unclean water, and poor sanitation and hygiene, and nutritional interventions have had a mixed effect on growth outcomes,^5^ and reductions in child mortality and diarrhoeal disease have not produced the expected decrease in stunting.^2^ Evidence from rural parts of The Gambia from the past 3 years suggests that major and sustained improvements in living conditions, disease reduction, diet, and health care might be needed to eliminate undernutrition.^2^, ^6^ The causal pathways remain poorly understood, precluding effective intervention.

We explore the role of temperature in growth faltering in sub-Saharan Africa. For thermeostasis, humans have two responses to heat stress: decreasing food intake to reduce metabolism and heat generation; and increasing heat loss—eg, through increased sweating, a higher area-to-mass ratio, and behavioural adaptations including reduced activity. In an earlier study,^7^ Wells hypothesised that these responses might contribute to growth faltering among children living in hotter climates via three mechanisms. First, decreasing food intake will reduce growth and enhance heat loss through an increased area-to-mass ratio. Second, prolonged heat exposure creates a selective pressure for reduced body size. Indeed, there is evidence that Bergmann's rule—that smaller organisms are found in warmer regions—might apply to modern humans where there is a difference between populations greater than 50 degrees of latitude, or 30°C, or both.^8^ Importantly, any effect on body size is likely to manifest as wasting (low weight-for-height), which increases area-to-mass ratio and cooling capacity, rather than stunting (low height-for-age), which has little effect on area-to-mass ratio.^7^ Third, heat stress incurs a high energetic cost—eg, due to increased sweating. Together, these effects are more pronounced in young children who have a poorer heat tolerance than adults because of their lower sweat rate and blood volume, among other factors.

**Research in context**

**Evidence before this study**

We searched PubMed between Jan 1, 1900, and Aug 1, 2019, and Embase between Jan 1, 1980, and Aug 1, 2019, and identified 45 unique studies with titles containing the terms (“temperature*”, “climate” OR “heat”) AND (“*nutrition”, “stunting”, “wasting”, “human growth”, “child growth”, “growth failure”, OR “growth faltering”). One modelling study proposed the hypothesis that child growth faltering is linked to high environmental temperatures, via an adaptive response to heat, reduced appetite, and the energetic cost of thermoregulation. The study recommended empirical investigation of this hypothesis, but we found no studies that did so. Five studies explored the relationship between environmental temperature and child growth, as mediated by food prices, income, or agriculture, but none investigated a direct biological effect.

**Added value of this study**

Our study is one of the first to consider a direct causal relationship between high environmental temperature and child growth faltering. We find evidence in support of this relationship in sub-Saharan Africa, with major potential implications for health. By analysing data for 656 107 children aged 0–5 years in 29 countries across sub-Saharan Africa, a region where growth faltering persists, we show that children living in hotter parts of sub-Saharan Africa are more likely to be wasted, underweight, and concurrently stunted and wasted, but less likely to be stunted, than in cooler areas.

**Implications of all the available evidence**

Child growth faltering remains a major source of morbidity and mortality in sub-Saharan Africa despite the scale-up of intensive nutrition, water and sanitation interventions, and widespread reductions in diarrhoea and all-cause mortality. To guide effective intervention, the missing causes of growth faltering urgently need to be understood. Our study suggests that increasing temperature in sub-Saharan Africa might increase the prevalence of wasting, underweight, and concurrent stunting and wasting, but decrease the prevalence of stunting. Future studies are needed to validate our findings, to investigate causal pathways, and to find strategies to mitigate against any effects of climate change on child growth.

We assessed whether exposure to high temperatures in sub-Saharan Africa is associated with child growth faltering. Our study is, to our knowledge, one of the first studies to explore a direct relationship between environmental temperature and early childhood growth outcomes. Our analysis is timely, given the predicted increase in global mean surface temperature by 0·3–4·8°C by the end of this century, relative to the late 20th century,^9^ and the urgent need to understand the effects of anthropogenic climate change on health.

## Methods

### Data sources

For growth outcomes in children aged 5 years or younger, data were extracted from the Demographic and Health Surveys (DHS) and Malaria Indicator Surveys (MIS).^10^ The DHS and MIS collect health and sociodemographic data using a two-stage random cluster sampling strategy, where clusters are randomly selected from census files and households are randomly selected in each cluster. We included all georeferenced DHS and MIS done in sub-Saharan Africa and available online by December, 2018, that collected anthropometric data and all prespecified covariables. Stunting was defined as a height-for-age Z score greater than two SDs below the reference median, wasting as a weight-for-height Z score less than –2, and underweight as a weight-for-age Z score of less than –2.

To assess health care and household characteristics, the following variables were extracted from DHS and MIS datasets for each child: age, sex, insecticide-treated net use the night before the survey, receipt of the third diphtheria-pertussis-tetanus (DPT-3) vaccination, receipt of the first measles vaccination (measles-1), any reported episode of diarrhoea in the 2 weeks before the survey, improved or unimproved drinking water source categorised using WHO Joint Monitoring Programme (WHO-JMP) criteria,^11^ improved or unimproved sanitation facility categorised using WHO-JMP criteria,^11^ education level of the household head, urban or rural residence, and floor type (finished [eg, parquet] *vs* unfinished or natural material [eg, earth]).

Household wealth index scores were calculated for each survey using linear principal component analysis (PCA). We applied inclusion criteria of fewer than 10% missing values and population frequency between 5% and 95% to the following assets: car, motorboat, scooter, cart, bicycle, television, refrigerator, radio, watch, mobile telephone, landline telephone, and electrification of the household. To condense these 12 assets into a single dimensional index we tested isometric mapping, kernel principle component analysis, t-distributed stochastic neighbour embedding, and linear PCA. We found minimal differences between algorithms, so used linear PCA because it is the most commonly used algorithm for wealth indices.^12^

For each georeferenced survey cluster the following data were extracted: synoptic mean monthly daytime land surface temperature (LST) from 2000 to 2016 (ie, the average temperature for each month derived from a multiyear time series);^13^ synoptic mean monthly enhanced vegetation index (EVI) from 2000 to 2016, a measure of the proportion of photosynthetically active radiation absorbed by vegetation, which is correlated with vegetation density and active photosynthesis;^13^ synoptic total monthly precipitation from 2000 to 2016;^14^ night-time lights, a proxy for regional wealth and urbanicity;^15^ and accessibility to large cities in 2015.^16^

### Data analysis

**Figure 1:**
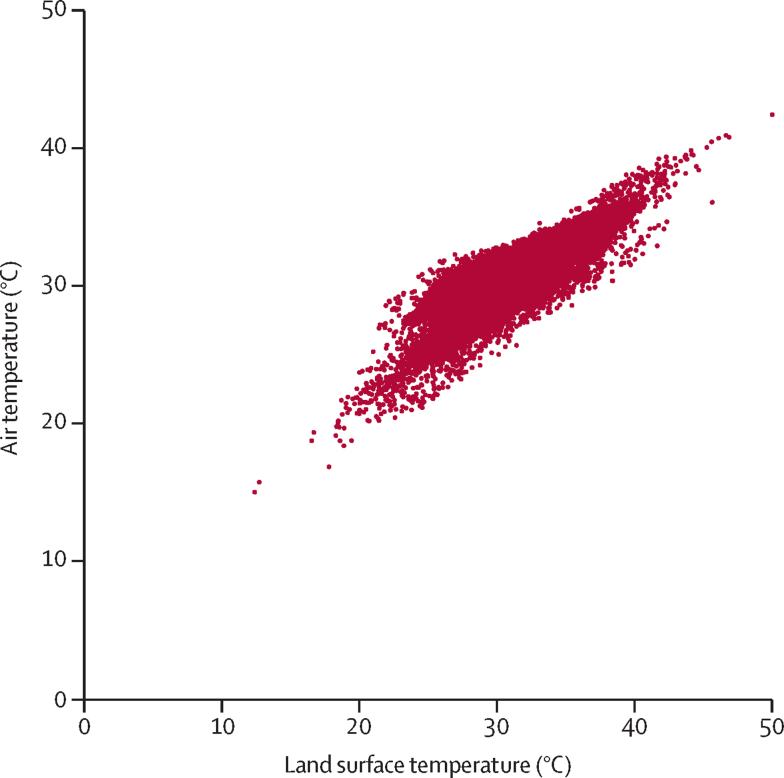
Monthly mean daytime air temperature versus monthly mean daytime land surface temperature for 25 089 georeferenced clusters in 52 surveys, 2000–16 Air temperature was determined from land surface temperature using a standard conversion.^17^

Logistic regression was used to estimate the odds of stunting, wasting, underweight, and concurrent stunting with wasting at the level of the child, relative to synoptic mean monthly daytime LST from 2000 to 2016 for the survey cluster. An LST of 30°C approximately translates into an air temperature of 27°C (figure 1),^17^ although this relationship is variable and depends on the month. The analysis controlled for survey, age, and sex of the child, insecticide-treated net use, DPT-3 vaccination, measles-1 vaccination, reported diarrhoea in the past 2 weeks, type of drinking water source, type of sanitation facility, attendance of the household head at secondary education, urban or rural survey cluster, type of household floor material, household wealth, monthly mean EVI, mean total monthly precipitation, night-time lights, and accessibility to large cities. Because growth outcomes vary with age,^18^ we stratified the analysis by age group (age <2 *vs* 2–5 years). To allow for a non-linear relationship between temperature and growth outcomes, LST was modelled as a categorical variable (ie, <30*°C*, 30°C to <35°C, and ≥35°C). These cutoffs were chosen to give a similar number of children in each temperature category. Because of the hierarchical nature of the dataset in which children were sampled within households and clusters, we used CIs that accounted for the highest level of clustering (ie, survey cluster) and we included survey as a fixed effect in the model.^19^ SEs were estimated using Taylor linearisation.

### Role of the funding source

The funder of the study had no role in study design, data collection, data analysis, data interpretation, or writing of the report. The corresponding author had full access to all the data in the study and had final responsibility for the decision to submit for publication.

## Results

**Figure 2:**
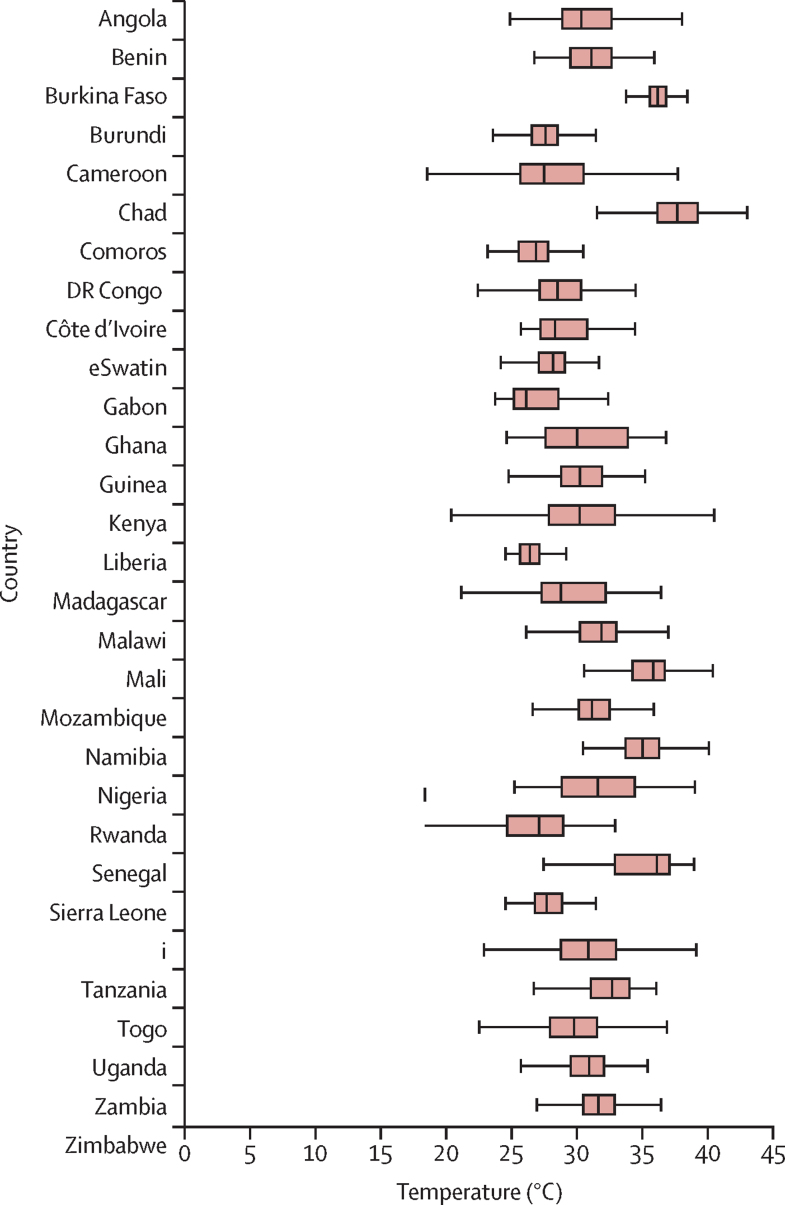
Monthly mean daytime land surface temperature for the 29 countries included in the analysis, 2000–16 (median, IQR, and range) Outside values are not shown.

Summary statistics are presented in the appendix (pp 1–2). Data were extracted for 121 DHS, MIS, and AIDS Indicator surveys. Of these, 52 DHS surveys that measured growth outcomes and all prespecified covariables were included in the multivariable analysis. The included surveys were done from 2003 to 2016, in 29 countries. The total study population comprised 656 107 children, aged 0–5 years, resident in 373 012 households. The median cluster size was 15 households (IQR 11–18). The mean age of children was 2·5 years (95% CI 2·5–2·5) and 325 919 (49·7%) of 656 096 children were female. During 2000–16, monthly mean LST in survey clusters ranged from a median of 26·1°C *(*IQR 25*·*2–28*·*5) in Gabon to 37*·*7°C *(*36*·*1–39*·*2) in Chad (figure 2).

Of the 336 299 children in 52 surveys for whom height and age were recorded, 102 213 (30·4%) were stunted. Stunting was more common among children aged 2–5 years (68 674 [33·5%] of 205 031) than children aged younger than 2 years (33 539 [25·6%] of 131 268; p<0·0001), and among boys (53 950 [31·9%] of 169 265) than girls (48 263 [28·9%] of 167 033; p<0·0001). Stunting prevalence ranged from 977 (15%) of 6637 in the Senegal 2016 DHS to 1758 (49%) of 3624 in the Burundi 2010 DHS (appendix pp 1–2). In the unadjusted analysis, the odds of stunting were 17% higher among children living in survey clusters where monthly mean daytime LST exceeded 35°C, compared with those in clusters where monthly mean daytime LST was less than 30°C (odds ratio [OR] 1·17, 95% CI 1·13–1·21; p<0·0001; appendix p 3). Controlling for the variables stated in the Methods section, the odds of stunting were 10% lower among children living in survey clusters where monthly mean daytime LST exceeded 35°C, compared with those in clusters where monthly mean daytime LST was less than 30°C (0·90, 0·85–0·96; p=0·00047; table). The reduction in stunting associated with an LST exceeding 35°C, compared with an LST of less than 30°C, was greater in children aged 2–5 years (0·89, 0·83–0·95; p=0·00081) than in children younger than 2 years (0·92, 0·85–0·99; p=0·034).

**Table:** Association between land surface temperature and growth outcomes in children aged 0–5 years in sub-Saharan Africa

	**Stunting**			**Wasting**			**Underweight**			**Stunting and wasting**		
	Prevalence, n (%)	OR* (95% CI)	p value	Prevalence, n (%)	OR* (95% CI)	p value	Prevalence, n (%)	OR* (95% CI)	p value	Prevalence, n (%)	OR* (95% CI)	p value
**All children**												
<30°C	96 687 (30·7%)	1 (ref)	0·0009	94 587 (5·9%)	1 (ref)	<0·0001	93 957 (19·4%)	1 (ref)	0·018	93 957 (1·4%)	1 (ref)	0·0058
30–34°C	106 552 (30·2%)	0·98 (0·94–1·01)	..	106 006 (7·9%)	1·05 (0·99–1·11)	..	104 995 (21·8%)	1·02 (0·98–1·06)	..	104 995 (2·0%)	1·17 (1·06–1·29)	..
≥35°C	66 668 (29·6%)	0·90 (0·85–0·96)	..	66 928 (12·5%)	1·27 (1·16–1·38)	..	66 343 (30·3%)	1·09 (1·02–1·16)	..	66 343 (3·6%)	1·23 (1·07-1·41)	..
**Children aged <2 years**												
<30°C	42 442 (25·8%)	1 (ref)	0·080	41 589 (7·9%)	1 (ref)	<0·0001	41 414 (18·5%)	1 (ref)	0·024	41 414 (1·7%)	1 (ref)	0·15
30–34°C	46 579 (25·9%)	0·98 (0·93–1·03)	..	46 197 (10·0%)	1·11 (1·04–1·19)	..	45 940 (20·5%)	1·07 (1·01–1·13)	..	45 940 (2·3%)	1·13 (0·99–1·28)	..
≥35°C	28 031 (24·0%)	0·92 (0·85–0·99)	..	27 986 (14·6%)	1·27 (1·15–1·40)	..	27 889 (26·7%)	1·11 (1·03–1·21)	..	27 889 (3·9%)	1·18 (0·98–1·41)	..
**Children aged 2–5 years**												
<30°C	54 245 (34·5%)	1 (ref)	0·0013	52 998 (4·4%)	1 (ref)	<0·0001	52 543 (20·2%)	1 (ref)	0·018	52 543 (1·2%)	1 (ref)	0·015
30–34°C	59 973 (33·5%)	0·98 (0·94–1·02)	..	59 809 (6·2%)	1·00 (0·92–1·09)	..	59 055 (22·8%)	0·99 (0·94–1·04)	..	59 055 (1·9%)	1·22 (1·06–1·41)	..
≥35°C	38 637 (33·7%)	0·89 (0·83–0·95)	..	38 942 (11·0%)	1·28 (1·13–1·45)	..	38 454 (33·0%)	1·07 (1·00–1·16)	..	38 454 (3·4%)	1·29 (1·06–1·57)	..

Temperatures are the monthly mean daytime land surface temperature, calculated from synoptic monthly means from 2000 to 2016. OR=odds ratio.

* Adjusted for survey, age and sex of the child, type of drinking water source, type of sanitation facility, third diphtheria-pertussis-tetanus immunisation, first measles immunisation, reported diarrhoea in the past 2 weeks, type of household floor material, insecticide-treated net use, attendance of household heads at secondary education, urban or rural survey cluster, household wealth, mean monthly enhanced vegetation index, mean monthly precipitation, urbanicity, night-time lights, and accessibility to large cities.

Of the 335 835 children in 51 surveys for whom weight and height were recorded, 26 264 (7·8%) were wasted. Wasting was more common among children younger than 2 years (13 351 [10·3%] of 130 054) than those aged 2–5 years (12 913 [6·3%] of 205 781; p<0·0001), and among boys (14 038 [8·3%] of 169 013) than girls (12 226 [7·3%] of 166 820; p<0·0001). Wasting prevalence ranged from 67 (2%) of 3828 children in the Rwanda 2015 DHS to 3897 (15%) of 26 463 in the Nigeria 2013 DHS. In the unadjusted analysis, the odds of wasting were 95% higher in countries where monthly mean daytime LST exceeded 35°C, compared with countries in which monthly mean daytime LST was less than 30°C (OR 1·95, 95% CI 1·85–2·05; p<0·0001; table). In the adjusted analysis, the odds of wasting were 27% higher in countries where monthly mean daytime LST exceeded 35°C, compared with those where monthly mean daytime LST was less than 30°C (1·27, 1·16–1·38; p<0·0001). The increase in wasting associated with LST exceeding 35°C was similar across age groups (age <2 years 1·27, 1·15–1·40, p<0·0001; age 2–5 years 1·28, 1·13–1·45, p=0·0001).

For the 330 809 children in 51 surveys for whom weight and age were recorded, 75 019 (22·7%) were underweight. Underweight was more common among children aged 2–5 years (47 587 [23·6%] of 201 562) than those younger than 2 years (27 432 [21·2%] of 129 247 children; p<0·0001) and among boys (38 887 [23·4%] of 166 487) than girls (36 132 [22·0%] of 164 321; p<0·0001). Underweight prevalence ranged from 203 (7%) of 2784 in the eSwatini 2006 DHS to 4017 (37%) of 10 749 in the Chad 2014 DHS. In the unadjusted analysis, the odds of underweight were 64% higher in countries where monthly mean daytime LST exceeded 35°C, compared with countries where monthly mean LST was less than 30°C (OR 1·64, 95% CI 1·57–1·70; p<0·0001; table). In the adjusted analysis, the odds of underweight were 9% higher among children living in survey clusters where monthly mean daytime LST exceeded 35°C, compared with children living in survey clusters where monthly mean daytime LST was less than 30°C (1·09, 1·02–1·16; p=0·0073). The increase in underweight associated with LST exceeding 35°C was slightly greater in children younger than 2 years (1·11, 1·03–1·21; p=0·011) compared with children aged 2–5 years (1·07, 1·00–1·16; p=0·063).

Height, age, and weight were recorded for 330 809 children in 51 surveys, of whom 6982 (2·1%) were both stunted and wasted. Concurrent stunting with wasting was more common in children younger than 2 years (3185 [2·5%] of 129 247) than those aged 2–5 years (3797 [1·9%] of 201 562; p<0·0001), and among boys (4039 [2·4%] of 166 487) than girls (2943 [1·8%] of 164 321; p<0·0001). The prevalence of concurrent stunting with wasting ranged from 27 (0·4%) of 6142 in the Zimbabwe 2015 DHS to 512 (5%) of 10 749 in the Chad 2014 DHS. In the unadjusted analysis, the odds of concurrent stunting with wasting were more than two times higher in countries where monthly mean daytime LST exceeded 35°C, compared with countries where monthly mean daytime LST was less than 30°C (OR 2·08, 95% CI 1·92–2·25; p<0·0001). In the adjusted analysis, the odds of concurrent stunting with wasting were 23% higher in countries where monthly mean daytime LST exceeded 35°C, compared with countries where monthly mean daytime LST was less than 30°C (1·23, 1·07–1·41; p=0·0037). The increase in concurrent stunting and wasting associated with LST exceeding 35°C was greater in children aged 2–5 years (1·29, 1·06–1·57; p=0·0099) compared with children aged younger than 2 years (1·18, 0·98–1·41; p=0·073).

## Discussion

We tested the hypothesis that young children in Africa exposed to high temperatures will experience growth faltering. By analysing data for children aged 0–5 years from 52 national surveys in sub-Saharan Africa, we found that monthly mean daytime LSTs exceeding 35°C were associated with a 27% increase in the odds of wasting, a 9% increase in the odds of underweight, and a 23% increase in concurrent stunting with wasting, but a 10% reduction in stunting, compared with temperatures averaging less than 30°C between 2000 and 2016. Previous studies of a direct effect of temperature on child growth are scarce. In a key study,^7^ Wells developed a model of the relationship between body size and heat production, showing that growth faltering can theoretically relieve heat stress in childhood and recommending empirical investigation of this hypothesis. Other studies^20^, ^21^ support a role for environmental temperature in early child growth, such as birthweight. For example, Wells and Cole^20^ observed, across 108 populations, that a one unit increase in heat index was associated with a 3% decrease in birthweight. To our knowledge, ours is the first empirical investigation of a direct relationship between temperature and early childhood growth outcomes.

High temperatures are hypothesised to contribute to growth faltering through three main mechanisms.^7^ First, wasting might be an adaptive response to high temperatures. Arguably it is harder to avoid heat stress in hot climates via behaviour change than in cooler climates, creating a greater reliance on physiological adaptations, particularly increased area-to-mass ratio.^7^ In the first 3 years of life, this pressure is exacerbated because the greater overall body fat relative to the weight of young children creates a low area-to-mass ratio and, compared with adults, young children have a lower sweat rate, greater blood volume per unit mass, higher metabolic rate per unit mass, and higher metabolic cost of locomotion, making it harder to keep cool.^22^ In addition the heat stress experienced in hot climates might be exacerbated in some African communities if a baby is swaddled, the young child is carried on its mother's back (appendix p 4), or the child sleeps in a metal-roofed house. In malnourished children, energy expenditure might be higher still, because organ tissue (which has a higher metabolic rate than normal tissue) is proportionally larger than in healthy children.^7^ Second, linked to physiological adaptation, appetite might be reduced in higher temperatures.^23^ If prolonged, lower food intake would contribute to growth faltering. Third, there is an energetic cost to keeping cool. Approximately 40–50% of total energy expenditure in mammals is used to maintain a constant core temperature and a 1°C increase in body temperature is associated with a 10–13% increase in metabolic rate.^24^ Children experiencing long-term heat stress might therefore allocate less energy to growth.

Increased temperatures were associated with a greater risk of wasting and underweight, but a decrease in stunting. Although future studies are needed to replicate this finding, several potential biological explanations exist. First, ambient temperature is known to modulate bone elongation in mammals, with limbs of animals raised in warmer ambient temperatures significantly and permanently longer than those of siblings housed at cooler temperatures.^25^ Thus, human limb length might increase with heat exposure. Second, if wasting is protective in high temperatures,^7^ stunted children might have a higher mortality rate than wasted children under heat stress. Wells’ model of temperature and body size suggests that stunting and wasting both affect surface area and energy expenditure, but stunting causes little increase in area-to-mass ratio in children aged 0–2 years.^7^ In contrast, wasting increases area-to-mass ratio by at least 15% in young children, promoting cooling capacity in early infancy. We found stunted children to be older than wasted children and the reduction in stunting associated with higher temperatures to be most pronounced among children aged 2–5 years. This finding is consistent with a decline in prevalence of stunted children in higher temperatures because of increased mortality, although research is needed to understand this further. There might be other, unknown, temperature-related drivers making wasting a more likely outcome of undernutrition than stunting as temperatures rise.

We hypothesise that a decline in stunting associated with increased temperatures might also be linked to genetic factors. Only 2% of our study population was both stunted and wasted, similar to a pooled prevalence of 3% in the same age group from a meta-analysis of 84 countries.^26^ This partly reflects the high mortality rate in children affected by both,^27^ but also illustrates how stunting and wasting largely affect different children. Indeed, a high prevalence of stunting can be observed in populations where wasting is uncommon.^28^ Human height is approximately 80% genetically determined by at least 600 genetic variants that are not yet fully understood,^29^ with Africans having the highest levels of within-population genetic diversity worldwide. It is possible that height in African populations is influenced by unknown epigenetic factors that interact with environmental temperature. Studies of the prevalence of stunting in relation to both ethnicity and environment in sub-Saharan Africa might be insightful.

We found that the relationship between temperature and growth outcomes varied with children's age, with the association between high temperature and growth faltering being greater among children aged 2–5 years than 0–2 years for stunting and concurrent stunting with wasting, and the same association smaller among children aged 2–5 years than 0–2 years for underweight. A previous study showed systematically larger estimates of association with risk factors for stunting in children aged 2–5 years than younger than 2 years, but systematically smaller effects in the older age group for wasting.^18^ This finding might be because wasting rates are generally highest in children aged younger than 2 years, but stunting can take longer to manifest and is more prevalent after 2 years.^18^ Here we observed a similar pattern. Our analysis shows the importance of age disaggregation in observational studies of child growth.

Previous studies investigating the causes of stunting, wasting, and underweight have focused mainly on nutrition, socioeconomic factors, exposure to infection and maternal health.^4^ Yet the persistence of growth faltering in sub-Saharan Africa despite the scale-up of nutrition and hygiene-based interventions suggests additional causes.^2^ The few studies^30^, ^31^ to have explored the role of climate have focused on the effect of environmental temperature on food security, rather than a direct biological effect, as we have done. Further research is needed to validate and understand our findings, but a direct, causal link between temperature and growth faltering would have major implications for public health. Mean annual temperatures are expected to rise by 2°C across Africa by the end of this century, relative to the late 20th century, with some scenarios showing rises of between 3°C and 6°C for the same period.^9^ Estimates of future deaths attributable to climate change, as well as public health strategies to mitigate against its effects might therefore need to consider growth faltering. Importantly, if wasting is an adaptive and arguably protective response to high temperatures, it might be helpful to counteract this effect by keeping children cool while ensuring adequate nutrition. Although research would be needed to identify appropriate interventions, one possible approach could be the promotion of cooler housing in hotter parts of sub-Saharan Africa. Rather than air conditioning, this can be achieved with buildings with low thermal mass and good ventilation.^32^

We observed an association between environmental temperature and child growth, but further research is needed to understand the extent to which this is mediated by food security and poverty, rather than a direct biological effect. Hotter climates are generally associated with greater levels of poverty, which is related to child growth. For example, Chad was the hottest country included in our analysis, but ranks near the lowest of the global Human Development Index.^33^ Despite controlling for local differences in wealth using a household-level wealth index, education level of the household head, urban–rural status and night-time lights, residual confounding is possible. Higher temperatures can also be associated with lower crop yields, affecting nutrition, and we attempted to control for this using EVI and precipitation. On the other hand, local climate is one of many factors influencing food security, which is also determined by food distribution, exchange and affordability, the resources available to prepare food safely, and the stability of such factors over time.^34^ Incorporating survey as a fixed effect in our model helped to account for national-level variation in such factors.

Our study has several limitations. First, our measure of temperature was based on synoptic means from 2000 to 2016, and is therefore an imprecise measure of climate conditions experienced by children of different ages and surveyed in different years. Nonetheless, child growth is a long-term process that would be expected to respond more slowly to environmental conditions than other health outcomes, such as vector-borne disease. Similarly, we used synoptic means for precipitation, and EVI and static estimates for night-time lights and accessibility. Second, LST does not directly translate into ambient temperatures, although it is the most precise metric of temperature available from remotely sensed data.^17^ Third, the OR, although a valid measure in its own right, might not be taken as approximation of the risk ratio when the outcome of interest is common (>10%). Fourth, as discussed, residual confounding by social and environmental factors is possible. Last, the causes of growth faltering are complex and our analysis is only a preliminary exploration of the role of temperature. Cross-sectional data, like all observational data, cannot definitively determine causality. However, because it is not feasible to randomise children to high or low temperatures, it is the only method available to investigate this question. Further studies are needed to confirm our findings, such as the observed decrease in stunting at higher temperatures. Research is also needed to understand causal pathways, including whether the persistence of growth faltering represents an adaption to hot climates, and to understand the relationship between heat exposure and growth over the human life course. Although we did not adjust for birthweight in our model, high environmental temperatures have previously been shown to be associated with lower birthweight^20^, ^21^ and shorter gestational length.^35^ Overall, we aim to highlight the potential role of environmental temperature in the growth of African children and to provide a stimulus for future research and discussion of this topic.

In conclusion, children aged 0–5 years living in areas of higher temperatures in sub-Saharan Africa are more likely to be wasted and underweight, but less likely to be stunted as compared with those in lower temperatures. Future studies should evaluate the relationship between heat exposure and growth outcomes in children and elucidate causal mechanisms. Any direct effect of temperature on early childhood growth would need to be considered in light of the likely increases in mean temperature that Africans will experience this century, caused by climate change.

## Data sharing

All health data used in this analysis are available to download free of charge by registered users from the Demographic and Health Surveys Program. Registration, data, and full dataset access instructions are available online. Remotely sensed data are available to download from the cited sources.

For **registration** see https://dhsprogram.com/data/new-user-registration.cfm. For data see https://dhsprogram.com/data/dataset_admin/download-manager.cfm.

For **full dataset access instructions** see https://dhsprogram.com/data/Using-DataSets-for-Analysis.cfm
