# Supplementary material for: Environmental temperature and growth faltering in African children: a cross-sectional study
Source: Lancet Planet Health. 2020 Mar 25;4(3):e116–23. doi: 10.1016/S2542-5196(20)30037-1 (PMC7232952; doi:10.1016/S2542-5196(20)30037-1)
Supplement: Supplementary appendix [file mmc1.pdf]

THE LANCET
Planetary Health

Supplementary appendix

This appendix formed part of the original submission and has been peer reviewed. We post it as supplied by the authors.

Supplement to: Tusting L S, Bradley J, Bhatt S, et al. Environmental temperature and growth faltering in African children: a cross-sectional study. *Lancet Planet Health* 2020: **4:** e116–23.

Supplementary Information

Table S1. Characteristics of surveys (n=52) included in the adjusted analysis

	Household-level characteristics						Child-level characteristics											
Survey	N	Household head attended secondary education (%)	Urban residence (%)	Finished floor material (%)	Improved drinking water source (%)	Improved sanitation (%)	N	Mean age in years (%)	Male (%)	Stunted (%, N)	Wasted (%, N)	Underweight (%, N)	Stunted and wasted (%, N)	Slept under ITN night before (%)	Received DPT-3 vaccine (%)	Received measles-1 vaccine (%)	Reported diarrhoea in past two weeks (%)
Angola 2015 DHS	16109	37.1	55.1	39.0	50.4	56.1	18311	2.5	49.6	31.6 (7453)	4.5 (7587)	23.0 (7453)	1.6 (7453)	21.1	30.1	39.5	14.2
Benin 2012 DHS	17422	20.4	40.8	58.9	77.2	29.1	17489	2.6	51.0	41.6 (8423)	14.7 (10486)	27.2 (8423)	3.0 (8423)	69.8	59.1	60.6	6.6
Burkina Faso 2010 DHS	14424	10.9	30.6	47.5	79.0	32.9	16969	2.4	50.9	29.5 (6878)	13.3 (6891)	29.6 (6878)	3.7 (6878)	48.5	79.2	71.6	15.1
Burundi 2010 DHS	8596	11.7	19.5	17.1	76.1	44.3	9025	2.4	50.3	48.5 (3624)	5.2 (3634)	32.6 (3624)	2.5 (3624)	46.3	88.1	80.3	25.0
Burundi 2016 DHS	15977	13.1	18.8	19.5	83.0	54.8	15544	2.5	50.3	47.5 (6355)	4.0 (6358)	34.9 (6355)	2.5 (6355)	38.7	86.2	69.1	21.6
Cameroon 2004 DHS	10462	35.9	46.3	50.0	32.0	35.3	9612	2.4	50.0	31.4 (3728)	4.6 (3843)	16.4 (3728)	1.3 (3728)	1.1	58.3	55.0	16.4
Cameroon 2011 DHS	14214	39.0	47.2	53.7	68.0	56.5	14276	2.4	49.7	27.8 (5923)	4.4 (6001)	16.9 (5923)	1.8 (5923)	13.2	63.9	62.0	20.5
Chad 2014 DHS	17233	15.7	22.5	8.4	53.9	12.5	22509	2.6	50.5	39.0 (10749)	12.3 (11057)	37.4 (10749)	4.8 (10749)	20.3	25.1	47.1	19.9
Comoros 2012 DHS	4482	32.5	42.2	65.9	89.3	38.2	3933	2.4	50.0	24.7 (2705)	10.1 (2727)	18.3 (2705)	1.8 (2705)	44.2	61.5	61.9	16.5
DRC 2007 DHS	8886	50.2	41.6	21.3	47.2	38.5	10227	2.4	49.4	39.6 (3630)	9.0 (3652)	29.1 (3630)	2.1 (3630)	12.3	45.2	57.0	16.7
DRC 2013 DHS	18171	52.8	29.9	14.3	40.3	37.6	22059	2.4	49.6	39.1 (8962)	6.9 (8979)	27.9 (8962)	2.1 (8962)	52.3	50.5	59.6	16.8
Cote d'Ivoire 2012 DHS	9686	22.1	41.4	77.2	79.0	45.5	9742	2.5	50.0	24.0 (3781)	6.3 (3844)	19.3 (3781)	1.4 (3781)	37.4	56.9	55.4	18.7
Gabon 2012 DHS	9755	50.2	54.8	68.3	80.4	38.8	7446	2.3	49.9	19.9 (4107)	3.3 (4142)	11.0 (4107)	0.7 (4107)	44.2	34.9	61.6	18.6
Ghana 2003 DHS	6251	47.8	40.3	84.1	66.0	32.6	4620	2.4	50.3	31.0 (3424)	7.6 (3451)	23.3 (3424)	2.1 (3424)	5.0	69.0	68.6	17.0
Ghana 2008 DHS	11778	55.8	43.9	81.7	77.6	65.4	7411	2.5	50.8	23.6 (2665)	8.4 (2677)	18.3 (2665)	1.2 (2665)	39.0	75.7	72.5	20.5
Ghana 2014 DHS	11835	57.6	50.2	91.5	65.9	68.0	7341	2.4	51.9	14.7 (3069)	4.8 (3074)	14.7 (3069)	1.1 (3069)	47.4	78.0	73.9	12.3
Guinea 2005 DHS	6282	14.1	27.3	42.1	30.0	26.4	7008	2.3	50.8	34.4 (2753)	9.1 (2895)	25.6 (2753)	2.9 (2753)	1.3	44.2	46.0	15.2
Guinea 2012 DHS	7109	19.3	35.2	55.3	73.4	45.0	8531	2.5	51.4	26.4 (3525)	9.7 (3531)	22.0 (3525)	2.2 (3525)	27.1	44.4	54.8	17.3
Kenya 2008 DHS	9057	34.4	32.1	45.0	64.3	50.0	7231	2.4	51.4	29.1 (5588)	7.1 (5634)	20.8 (5588)	1.9 (5588)	52.2	72.4	68.8	17.1
Kenya 2014 DHS	36430	33.5	38.2	43.8	64.5	47.9	26253	2.5	50.6	21.5 (20699)	5.2 (20793)	17.4 (20699)	1.4 (20699)	52.4	81.5	72.4	15.0
Liberia 2013 DHS	9333	40.4	37.0	36.4	64.9	33.6	9724	2.5	51.2	27.7 (3877)	5.9 (3880)	20.2 (3877)	1.3 (3877)	36.8	55.1	58.0	25.2
Madagascar 2008 DHS	17857	29.1	25.2	29.8	43.8	7.4	15763	2.5	50.6	43.4 (5490)	-	-	-	46.4	65.3	59.9	8.8
Malawi 2010 DHS	24825	19.9	11.7	19.9	79.8	11.3	24280	2.5	49.4	40.6 (4927)	3.7 (4935)	17.4 (4927)	1.0 (4927)	42.8	85.3	78.8	17.2
Malawi 2015 DHS	26361	27.6	18.9	28.3	87.0	83.3	21414	2.6	49.9	28.6 (5677)	2.5 (5719)	16.1 (5677)	0.8 (5677)	43.8	82.1	68.4	21.1
Mali 2006 DHS	12998	10.3	31.8	22.6	55.2	21.8	15687	2.4	50.6	34.4 (11785)	13.6 (11994)	31.9 (11785)	4.1 (11785)	35.3	56.3	56.1	12.0

Survey	Household-level characteristics						Child-level characteristics										
	N	Household head attended secondary education (%)	Urban residence (%)	Finished floor material (%)	Improved drinking water source (%)	Improved sanitation (%)	N	Mean age in years (%)	Male (%)	Stunted (%, N)	Wasted (%, N)	Underweight (%, N)	Stunted and wasted (%, N)	Slept under ITN night before (%)	Received DPT-3 vaccine (%)	Received measles-1 vaccine (%)	Reported diarrhoea in past two weeks (%)
Mali 2012 DHS	10107	14.1	27.4	28.3	67.9	43.8	12882	2.5	50.9	33.0 (4861)	11.6 (4888)	30.3 (4861)	3.3 (4861)	68.2	55.1	61.1	9.0
Mozambique 2011 DHS	13919	17.2	36.6	32.5	58.3	22.9	12683	2.4	49.9	33.7 (10302)	4.2 (10413)	17.0 (10302)	0.9 (10302)	33.1	72.6	71.6	10.7
Namibia 2006 DHS	9200	44.4	42.3	53.1	88.1	44.0	6774	2.4	49.8	24.4 (5156)	7.0 (5206)	21.9 (5156)	2.0 (5156)	10.9	77.1	69.7	13.8
Namibia 2013 DHS	9849	53.9	48.4	60.2	87.4	47.9	6953	2.5	49.5	19.4 (2589)	6.7 (2619)	17.3 (2589)	1.5 (2589)	6.1	77.5	73.5	19.5
Nigeria 2008 DHS	34070	38.1	31.5	59.8	52.6	49.0	31634	2.4	50.9	38.3 (20862)	12.8 (20936)	28.3 (20862)	3.4 (20862)	6.1	30.1	36.0	10.8
Nigeria 2013 DHS	38522	42.7	41.2	65.4	58.3	52.6	35364	2.4	50.7	32.3 (26424)	14.7 (26463)	31.0 (26424)	3.8 (26424)	18.2	36.7	38.0	10.6
Rwanda 2010 DHS	12540	11.7	16.0	18.3	74.2	75.0	10697	2.6	50.8	36.6 (4380)	2.5 (4396)	15.3 (4380)	0.8 (4380)	67.8	91.2	81.6	13.4
Rwanda 2015 DHS	12698	13.6	22.8	26.6	74.2	71.7	9505	2.5	50.5	32.0 (3811)	1.8 (3828)	12.0 (3811)	0.6 (3811)	65.9	91.8	30.9	12.3
Senegal 2005 DHS	7412	12.1	42.0	60.0	63.8	40.5	13684	2.4	51.1	17.8 (3165)	7.9 (3246)	18.7 (3165)	1.9 (3165)	11.5	67.0	61.7	22.0
Senegal 2010 DHS	7904	11.5	37.5	54.7	69.5	44.3	15752	2.5	51.4	24.9 (3953)	8.6 (4306)	24.8 (3953)	2.1 (3953)	44.0	73.4	71.1	19.6
Senegal 2012 DHS	4177	12.8	39.2	66.0	66.9	51.3	8746	2.5	50.0	16.6 (6497)	9.1 (6636)	22.5 (6497)	2.2 (6497)	48.6	79.4	68.0	15.5
Senegal 2014 DHS	4233	11.5	39.1	62.9	69.5	49.1	8432	2.4	50.2	17.1 (6714)	6.4 (6807)	19.6 (6714)	1.6 (6714)	49.2	79.9	68.7	20.4
Senegal 2015 DHS	4511	12.0	38.9	70.3	65.5	49.1	8553	2.4	49.9	17.3 (6818)	7.6 (6877)	21.8 (6818)	1.8 (6818)	56.3	79.7	66.5	21.5
Senegal 2016 DHS	4440	12.5	39.0	69.6	72.0	52.5	8380	2.5	51.2	14.7 (6637)	7.2 (6714)	20.0 (6637)	1.6 (6637)	67.4	79.9	66.4	17.2
Sierra Leone 2008 DHS	7284	26.0	40.6	39.9	55.2	46.7	7426	2.4	50.1	31.9 (2648)	9.4 (2680)	24.2 (2648)	2.0 (2648)	27.3	51.6	51.4	12.2
Sierra Leone 2013 DHS	12629	24.6	36.2	41.5	58.8	50.0	14958	2.6	49.5	32.1 (5104)	7.9 (5143)	20.4 (5104)	2.0 (5104)	49.8	68.5	70.7	12.0
Swaziland 2006 DHS	4843	44.6	38.8	89.0	71.5	41.2	3713	2.5	49.4	23.8 (2784)	2.3 (2820)	7.3 (2784)	0.5 (2784)	0.8	84.8	76.2	15.1
Tanzania 2010 DHS	9623	14.0	23.0	33.8	52.4	25.5	10107	2.5	49.5	34.0 (7605)	5.5 (7611)	22.1 (7605)	1.4 (7605)	61.3	80.7	71.1	14.1
Tanzania 2015 DHS	12563	18.7	28.9	-	63.9	36.7	12745	2.4	49.9	27.3 (10222)	4.3 (10234)	18.2 (10222)	1.3 (10222)	49.8	76.8	61.6	12.1
Togo 2013 DHS	9549	36.8	38.1	85.5	63.2	38.3	8583	2.5	50.4	23.5 (3531)	6.3 (3541)	21.3 (3531)	1.9 (3531)	43.4	76.2	66.5	16.3
Uganda 2006 DHS	8870	22.7	15.7	22.6	69.3	27.1	10064	2.5	49.1	33.0 (2670)	5.4 (2715)	21.5 (2670)	1.9 (2670)	11.9	22.8	62.7	27.0
Uganda 2016 DHS	19588	30.8	22.8	33.4	77.0	34.4	19453	2.6	50.4	23.5 (5180)	3.2 (5266)	13.6 (5180)	1.0 (5180)	58.5	69.7	59.4	21.0
Zambia 2007 DHS	7164	37.9	37.6	36.7	42.5	33.0	7404	2.3	49.3	38.2 (5639)	5.2 (5647)	19.2 (5639)	1.0 (5639)	32.9	34.0	70.9	16.0
Zambia 2013 DHS	15920	44.5	43.7	41.9	62.1	41.5	16657	2.5	50.6	33.7 (12474)	5.5 (12495)	19.7 (12474)	1.1 (12474)	40.0	80.4	74.0	16.5
Zimbabwe 2005 DHS	9285	48.9	32.9	66.5	77.3	63.4	7284	2.6	50.4	29.0 (4979)	6.2 (5008)	16.4 (4979)	1.5 (4979)	3.1	52.7	52.2	13.1
Zimbabwe 2010 DHS	9756	53.5	34.1	69.8	78.5	63.1	7187	2.4	50.2	25.9 (5355)	2.9 (5414)	13.6 (5355)	0.8 (5355)	10.6	65.4	65.1	13.8
Zimbabwe 2015 DHS	10534	61.7	41.2	76.6	81.6	69.7	8082	2.6	49.6	20.4 (6142)	3.0 (6142)	11.1 (6142)	0.4 (6142)	9.2	75.1	61.0	16.9

ITN: insecticide-treated bednet; DPT: diphtheria-pertussis-tetanus; DRC: Democratic Republic of the Congo.

Table S2. Association between land surface temperature and growth outcomes in children aged 0-5 years in sub-Saharan Africa (unadjusted analysis)

Land surface temperature[a]	Stunting			Wasting			Underweight			Stunting and wasting		
	Prevalence (N)	OR[b] (95% CI)	p	Prevalence (N)	OR[b] (95% CI)	p	Prevalence (N)	OR[b] (95% CI)	p	Prevalence (N)	OR[b] (95% CI)	p
**All children**												
<30°C	32.5 (156,782)	1		5.6 (155,552)	1		20.3 (153,619)	1		1.5 (153,619)	1	
30°C to 34°C	32.1 (178,317)	1.03 (1.01, 1.06)	<0.0001	7.3 (179,847)	1.27 (1.22, 1.32)	<0.0001	22.4 (176,497)	1.17 (1.14, 1.20)	<0.0001	2.0 (176,497)	1.39 (1.30, 1.48)	<0.0001
≥35°C	31.2 (101,374)	1.17 (1.13, 1.21)		12.7 (102,773)	1.95 (1.85, 2.05)		31.2 (100,988)	1.64 (1.57, 1.70)		3.9 (100,988)	2.08 (1.92, 2.25)	
**Children aged <2 years**												
<30°C	26.5 (61,283)	1		7.9 (60,427)	1		19.0 (60,142)	1		1.8 (60,142)	1	
30°C to 34°C	27.4 (70,914)	1.07 (1.04, 1.11)	<0.0001	10.1 (70,755)	1.22 (1.17, 1.28)	<0.0001	21.6 (70,229)	1.22 (1.17, 1.26)	<0.0001	2.5 (70,229)	1.37 (1.26, 1.50)	<0.0001
≥35°C	24.8 (39,853)	1.17 (1.12, 1.23)		15.6 (39,895)	1.64 (1.54, 1.74)		27.8 (39,698)	1.55 (1.48. 1.63)		4.2 (39,698)	1.84 (1.65, 2.04)	
**Children aged 2-5 years**												
<30°C	36.3 (95,499)	1		4.1 (95,125)	1		21.2 (93,477)	1		1.2 (93,477)	1	
30°C to 34°C	35.2 (107,403)	1.01 (0.98, 1.04)	<0.0001	5.6 (109,092)	1.32 (1.24, 1.40)	<0.0001	22.9 (106,268)	1.14 (1.10, 1.18)	<0.0001	1.7 (106,268)	1.39 (1.27, 1.52)	<0.0001
≥35°C	35.3 (61,521)	1.17 (1.12, 1.22)		10.7 (62,878)	2.35 (2.18, 2.54)		33.3 (61,290)	1.68 (1.61, 1.76)		3.7 (61,290)	2.31 (2.08, 2.57)	

[a] Monthly mean daytime land surface temperature, calculated from synoptic monthly means from 2000 to 2016

[b] Adjusted for survey

OR: Odds Ratio; CI: confidence interval

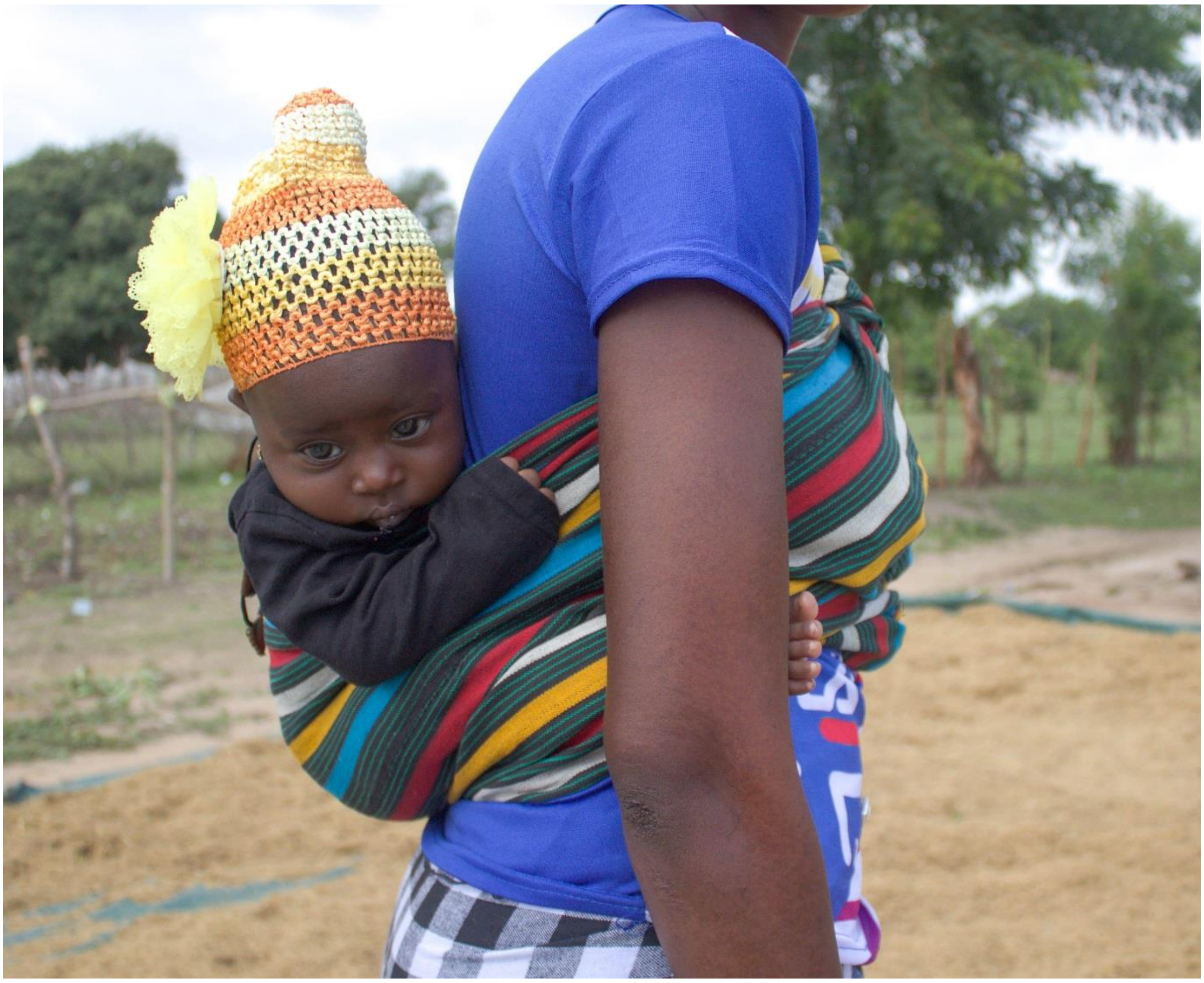

Figure S1. Child carried on her mother's back in The Gambia. Back-carrying of young children is common across sub-Saharan Africa. Photograph: Maria J. Carrasco-Tenezaca.
